# Diagnostic Accuracy of Contrast-Enhanced FLAIR Magnetic Resonance Imaging in Diagnosis of Meningitis Correlated with CSF Analysis

**DOI:** 10.1155/2014/578986

**Published:** 2014-03-20

**Authors:** Aneel Kumar Vaswani, Waseem Mehmood Nizamani, Muhammad Ali, Geeta Aneel, Bhesham Kumar Shahani, Sajjad Hussain

**Affiliations:** ^1^Dr. Ziauddin Hospital, Block 6, Scheme 5, Clifton, Karachi 74700, Pakistan; ^2^Dr. Ziauddin Hospital, Block B, North Nazimabad, Karachi 74700, Pakistan

## Abstract

*Purpose*. To determine the diagnostic accuracy of contrast enhanced FLAIR sequence of MRI brain in the diagnosis of meningitis. *Subjects and Methods*. A prospective study of 57 patients with signs and symptoms of meningitis, referred to the radiology department for MRI examination. Out of these, there were 30 males and 27 females. They underwent MRI brain with contrast including postcontrast T1W and FLAIR sequences. Cerebrospinal fluid (CSF) analysis obtained by lumbar puncture after MRI was considered the “reference standard” against which MRI findings were compared. *Results*. Of 57 patients, 50 were diagnosed as having meningitis on subsequent CSF analysis. Out of these 50, 49 were positive on postcontrast FLAIR images and 34 were positive on postcontrast T1W images. One patient was labeled false positive as CSF analysis showed malignant cells (leptomeningeal carcinomatosis). In the diagnosis of meningitis, the sensitivity of postcontrast FLAIR sequence was 96% and specificity 85.71%, whereas the sensitivity of postcontrast T1W sequence was 68% and specificity 85.71%. *Conclusion*. Contrast-enhanced FLAIR sequence is more sensitive and specific than contrast-enhanced T1W sequence in the diagnosis of meningitis. It should be routinely used in suspected cases of meningitis.

## 1. Introduction

Meningitis continues to be an important disease throughout the world and can be a life-threatening emergency if not suspected, appropriately diagnosed, and managed expeditiously [1]. Meningitis prevails to be one of the most serious causes of hospital admission in Pakistan in all age groups [2]. Estimated prevalence of meningitis in our region is 1.57% [3]. Delay in administration of antibiotics is associated with death in adults suffering from acute bacterial meningitis. A delay of 4–6 hours in the administration of antibiotics after presentation independently conferred an 8.4-fold greater risk of death from meningitis [4]. Infective meningitis including tuberculous and bacterial meningitis is the leading cause of stroke in young patients in our country [5]. Bacterial meningitis is the major cause of morbidity in children below the age of 5 years [6].

The diagnosis of meningitis is established by history, physical examination, and laboratory evaluation but the ability to detect and differentiate intracranial infections has markedly improved with the introduction of MRI. The lack of bone artifacts and the multiplanar capability of MRI have led to this preeminence [7]. Computed tomography and magnetic resonance imaging play important roles in diagnosing brain infections but magnetic resonance imaging is more accurate. Therefore, it is recommended as a first line diagnostic tool in brain infections [8].

Gd-DTPA-enhanced MR images were superior to postcontrast CT scans, not only in the detection of meningeal involvement but also in the identification of complications. MR is also superior in its ability to detect extracerebral fluid collections since it is free of bony artifacts adjacent to the inner table of the skull. The major role for Gd-DTPA in meningitis likely will be the identification of active blood/brain barrier disruption and increased vascularity, possibly facilitating the detection of the disease process at an early stage when it may not be detected on CT. Thus, if Gd-DTPA is used, MR appears to be superior to CT in the evaluation of leptomeningitis [9].

Contrast-enhanced FLAIR images have been shown to be superior to contrast-enhanced T1-weighted images in visualization of inflammatory leptomeningeal disease. Leptomeningeal disease can be more easily visualized on contrast-enhanced FLAIR images than on contrast-enhanced T1-weighted images because FLAIR imaging allows for a clearer distinction between enhancing meninges and enhancing cortical veins, cortical veins becoming less clearly enhanced on FLAIR images [10].

Splendiani et al. [11] stated that although conventional contrast-enhanced T1-weighted SE sequences are largely used in the diagnosis of many pathological conditions of the CNS, in their experience these sequences show a relatively low sensitivity (50%) with respect to contrast-enhanced FLAIR sequences (100%) for infectious meningitis.

To our knowledge no local data focusing on gadolinium-enhanced FLAIR sensitivity in early diagnosis of infectious meningitis is available. Hence, by conducting this study, we would know the significance of contrast-enhanced FLAIR for the early diagnosis of infectious meningitis in our setup as compared to the international literature [7, 10, 11]. Early diagnosis through contrast-enhanced FLAIR MRI will be helpful in the early and effective treatment, will reduce morbidity and mortality, and will offer the patients a better prognosis.

## 2. Materials and Methods

Over a period of  1 year, 57 patients who presented with clinical suspicion of meningitis were evaluated. This was a cross-sectional, validation study. The sample technique used was nonprobability, purposive type.

Patients referred to the radiology department for magnetic resonance imaging with clinical symptoms of meningitis such as severe headache, fever, neck stiffness, photophobia, nausea, vomiting, and altered consciousness were included in the study.

Informed consent was taken from all the patients after explaining the study's purpose, procedure, and risk-benefit ratio and approval of the ethical committee was given. History was taken in each case. Patients who had started prophylactic antibiotics and who were already known cases of meningitis were excluded from the study in order to control confounding variables.

MRI scan of brain from the vertex till the base of skull was performed by 1.5 tesla (T) Toshiba Exelart Model MRT-1501/P3scanner. Contrast-enhanced T1W and contrast-enhanced FLAIR images were acquired. Imaging parameters of contrast-enhanced T1W imaging were TR: 500, TE: 7.8, FOV: 230 mm, image matrix: 224 × 256, slice thickness: 5 mm, slice interval: 1.5 mm, phase encoding direction, and R to L and acquisition time: 3 min 48 seconds. Imaging parameters of contrast-enhanced FLAIR imaging were TR: 9000, TE: 109, TI: 2500, FOV: 230 mm, image matrix: 224 × 256, slice thickness: 5 mm, slice interval: 1.5 mm, phase encoding direction, and R to L and acquisition time: 2 min 08 seconds. All patients received intravenous gadolinium contrast medium (the dose of which was decided according to weight of the patient) given by a computer-controlled injector at rate of 0.2 mL/second.

The MRI images of postcontrast FLAIR and postcontrast T1W sequences were evaluated by simple visual inspection by experienced radiologist and attention was paid to determine the presence or absence and the location and the extension of pathological altered leptomeningeal signals. The subarachnoid space (SAS) was considered abnormal for gadolinium-enhanced T1W and FLAIR if the reader found abnormal enhancement in sulci, cisterns, ventricles, or any combination of these. This data was then recorded on proforma by the researcher as positive or negative.

Final diagnosis of meningitis was obtained by CSF analysis results obtained from the laboratory.

Total protein less than 0.45 g/L, glucose ratio greater than 0.4-0.5 mmoL/L, and lactate less than 1.0–2.9 are normal CSF biochemical values. Cell count less than 15 per 3.2 µL, mononuclear cells on cytology, negative culture, and serology are normal findings [12].

The statistical analysis was done using SPSS windows package version 22.0. Descriptive analysis was conducted, that is, frequencies and percentages for categorical variables like gender, mean, and standard deviation for the continuous variables like age. Frequency was calculated in terms of presence or absence of meningitis on contrast-enhanced T1W images and contrast-enhanced FLAIR images out of total cases.* P* value of equal to or less than 0.05 was considered significant. Sensitivity, specificity, and negative and positive predictive values were determined by taking CSF analysis as reference standard.

## 3. Results

Initially during recruitment period, 65 patients presented with clinical suspicion of meningitis. Out of these, 8 patients were excluded from the study because two patients were already diagnosed as having meningitis, 3 patients had noncompatible metallic implants (two of whom had cardiac pace-makers and other had cochlear implant), 1 patient had already taken antibiotics, and 1 patient was claustrophobic and contrast agent was contraindicated in 1 patient. Therefore, the final number of patients comprising the study was 57, who underwent MRI for clinical suspicion of meningitis. Out of 57 patients, 30 patients (52.6%) were male and 27 patients (47.4%) were female. The mean age was 30.65 ± SD 21.25 years ranging from 1 month to 75 years.

The presence or absence of signs and symptoms such as headache, fever, neck rigidity, vomiting, photophobia, and Kerning's and Brudzinski's signs was documented. The most frequently occurring symptom in our study was vomiting, observed in 90% of patients. The other most occurring symptoms were headache and fever identified in 88.7% and 86.2% of patients, respectively. The other less frequently recorded signs and symptoms were neck rigidity, Kerning's sign, Brudzinski's sign, and photophobia.

**Figure 1:**
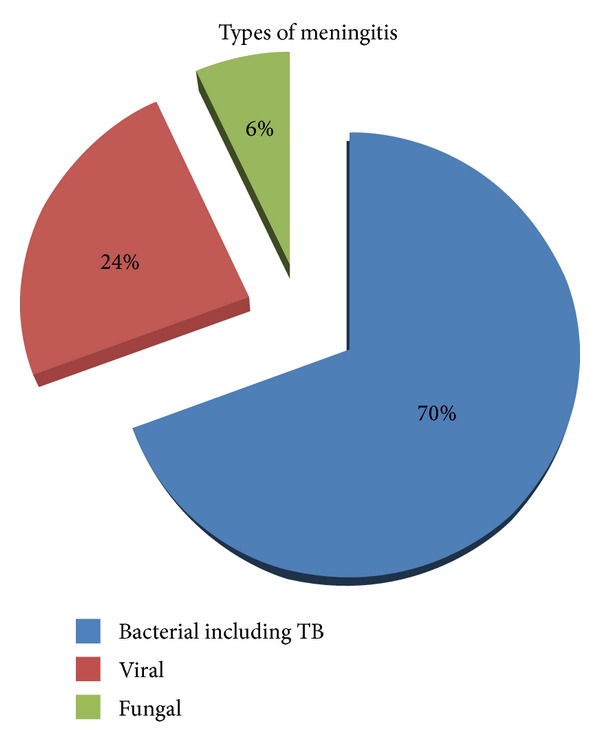
Pie chart showing percentages of etiological organisms causing meningitis in patients included in this study.

After MRI examination, each patient underwent a lumber puncture for CSF analysis to confirm the diagnosis. Out of 57 patients, 50 patients (87.71%) had CSF positive meningitis and 1 patient showed malignant cells on CSF analysis and was also positive on postcontrast MR examination (false positive). Remaining 6 patients were true negative. Out of 50, 35 cases (70%) had bacterial (including tuberculous) meningitis, 12 cases (24%) had viral meningitis, and three cases (6%) had fungal meningitis (Figure 1).

The analysis of unenhanced images did not demonstrate an altered signal on T1-weighted or T2-weighted images but two cases showed meningeal hyperintensities on plain FLAIR images. As contrast-enhanced images are included in the evaluation, 49 patients (96%) showed pathological meningeal enhancement at MRI examination and two patients (3.9%) had normal MRI.

In 35 cases (70%), the meningeal enhancement was observed in both contrast-enhanced T1-weighted and FLAIR sequences and in 14 cases (28%) enhancement was only demonstrated on postcontrast FLAIR sequence.

**Figure 2:**
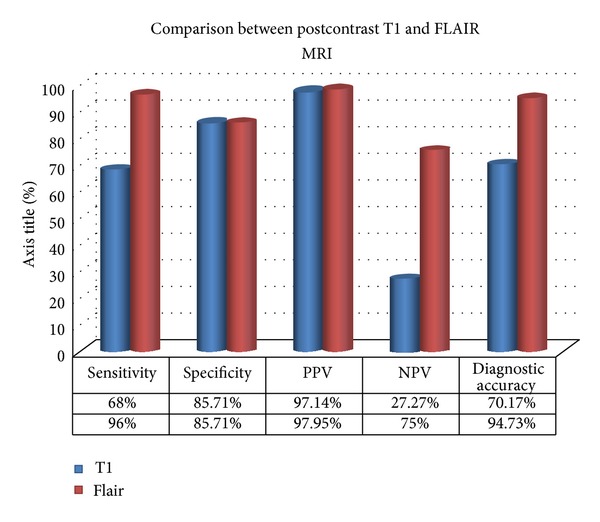
Bar chart showing comparison of this study results between postcontrast T1 and FLAIR MRI.

After CSF results, the sensitivity of contrast-enhanced FLAIR sequences was 96%, specificity 85.71%, positive predictive value 97.95%, and negative predictive value 75%, while contrast-enhanced T1-weighted sequences showed a sensitivity of 68.%, specificity of 85.71%, positive predictive value of 97.14%, and negative predictive value of 27.27% (Figure 2). In comparison with postcontrast T1, the meningeal enhancement on postcontrast FLAIR was more extensive (Cases 1–3).

*Cases of the Study*

*Case Number 1*. 35-year-old male patient presented with high-grade and vomiting. See Figures 4 and 5.

*Case Number 2*. 35-year-old female patient presented with the history of fever and neck rigidity. See Figures 6 and 7.

*Case Number 3*. 55-year-old man presented with the history of severe headache and vomiting. See Figures 8 and 9.

Concerning etiology, no specific findings were registered on MRI to differentiate between viral, bacterial, or fungal meningitis. However, the meningeal enhancement was located in basal and subarachnoid cisterns in tuberculous and fungal meningitis whereas, in bacterial meningitis, the enhancement was located over the cerebral convexity and along sylvian fissures. Six patients also had parenchymal changes like cerebritis and tuberculomas that appeared as focal hyperintense parenchymal signals with postcontrast enhancement.

McNemar test was applied to compare the frequency of detection of meningitis by contrast-enhanced FLAIR and contrast-enhanced T1WI to see statistical significance at 95 % confidence interval which gave *P* value of 0.01 (*P* = 0.01). Therefore, we have sufficient evidence to conclude that contrast-enhanced FLAIR is superior to contrast-enhanced T1WI for diagnosis of meningitis.

## 4. Discussion

Conventional sequences have routinely been used in diagnosis of meningitis, such as postcontrast T1-weighted images. Contrast-enhanced FLAIR images were shown to be better than contrast-enhanced T1-weighted images in visualization of inflamed meninges. Leptomeningeal disease can be more easily visualized on contrast-enhanced FLAIR images than on contrast-enhanced T1-weighted images because FLAIR imaging allows for a better distinction between enhancing meninges and enhancing cortical veins, cortical veins being enhanced less on FLAIR images [18–20].

**Figure 3:**
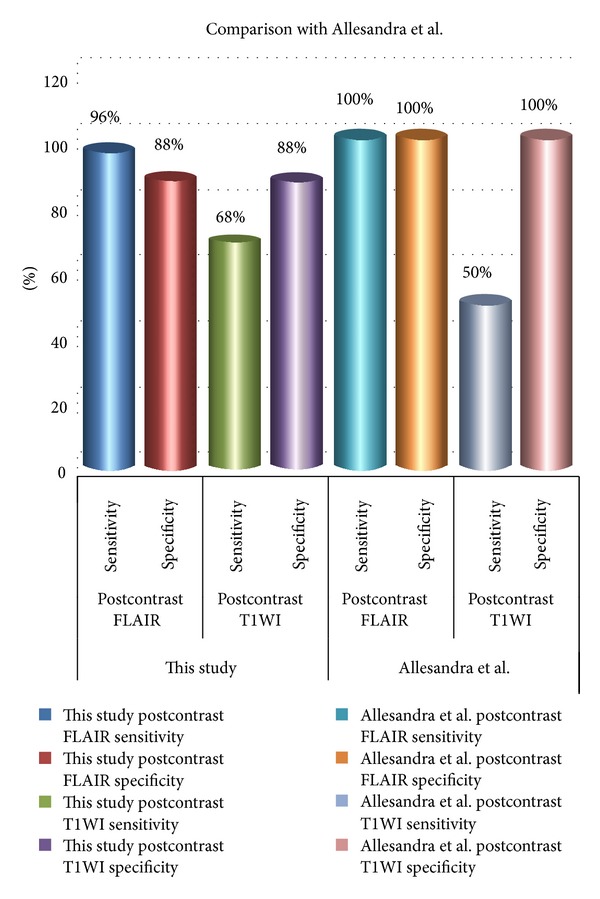
Bar chart showing comparison of this study results with Allesandra et al. results.

**Figure 4:**
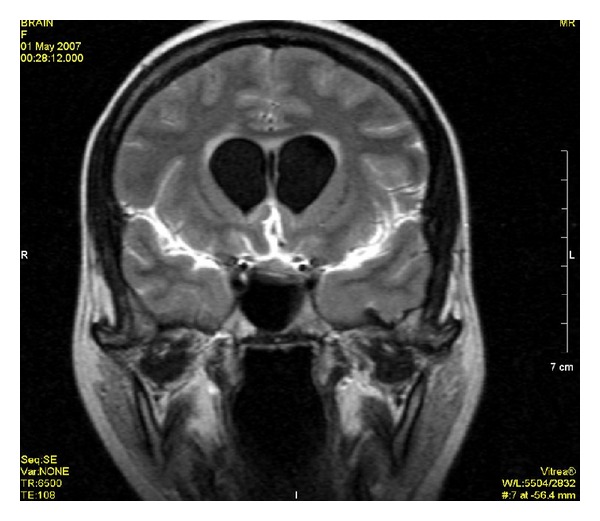
Postcontrast FLAIR image shows the enhancement of meninges at tentorium and in parietal region with evidence of dilated ventricles.

**Figure 5:**
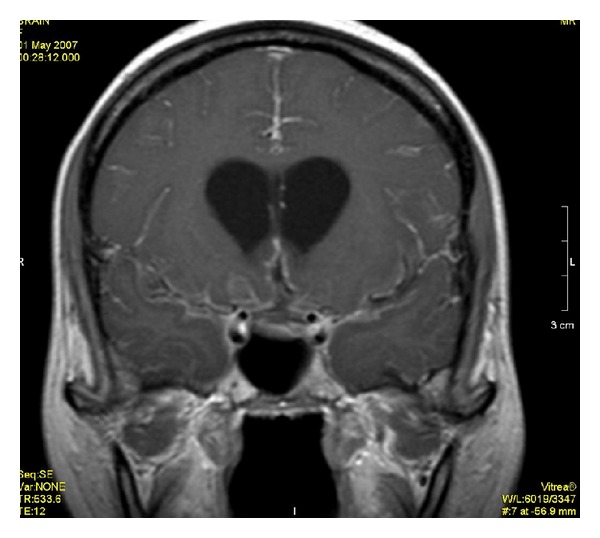
Postcontrast T1WI shows only dilated ventricles with no evidence of meningeal enhancement.

**Figure 6:**
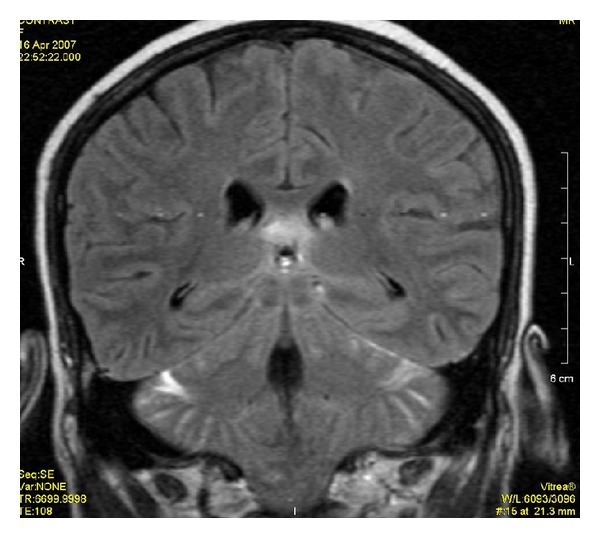
Postcontrast FLAIR image shows meningeal enhancement along cerebellar cortex and along left tentorium.

**Figure 7:**
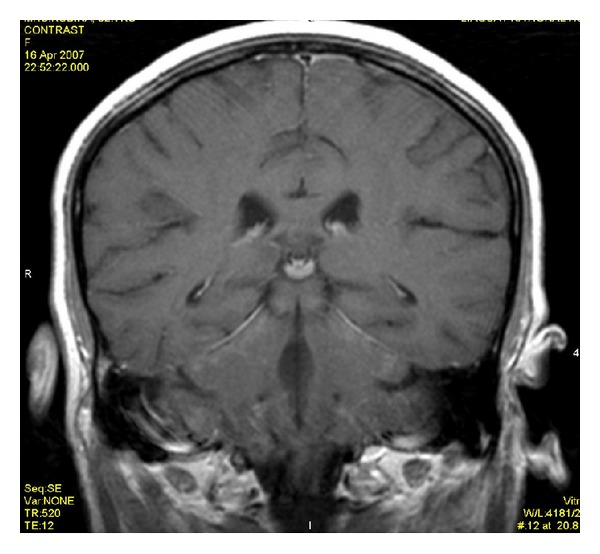
Postcontrast T1WI does not show meningeal enhancement.

**Figure 8:**
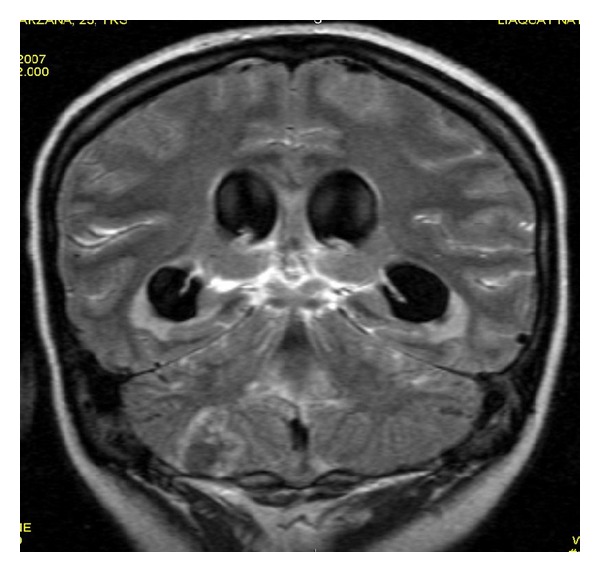
Postcontrast FLAIR image shows patchy areas of meningeal enhancement with dilated ventricles and irregular ring enhancing lesion representing postmeningitis abscess formation.

**Figure 9:**
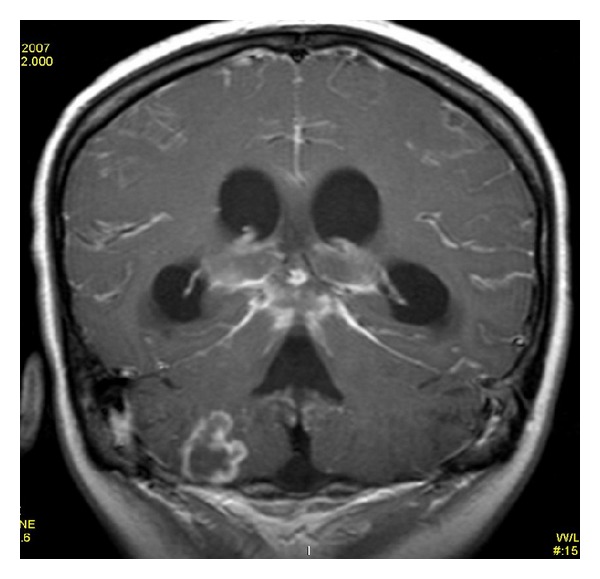
Postcontrast T1WI shows same findings as in figure number 10 but less extensive.

The results of contrast-enhanced FLAIR sequence, in this study, are almost comparable to Allesandra et al.. Out of the total 12 patients they included in their study, contrast-enhanced FLAIR was positive in all cases [11]. However, contrast-enhanced T1-weighted images diagnosed meningitis in only 6 patients which is only 50%, whereas, in our study, contrast-enhanced T1 diagnosed 68% of cases. The difference in results could be due to difference in technique or it could be due to the number of patients included in the study. However, the hypothesis of both studies is similar concluding that contrast-enhanced FLAIR is a much better sequence in diagnosing meningitis as compared to contrast-enhanced T1-weighted images (Figure 3).

In 2006, Parmar and his colleagues [7] conducted a study to determine contrast-enhanced FLAIR in the evaluation of infectious leptomeningeal diseases and suggested that postcontrast FLAIR images have similar sensitivity but a higher specificity compared to contrast-enhanced T1-weighted images for detection of leptomeningeal enhancement. It can be a useful adjunct to postcontrast T1-weighted images in evaluation of infectious leptomeningitis. The age group in their study was 3–78 years, which is comparable to our study. In our study, some patients were less than 3 years, youngest being 3 months. The reason is that in our population vertical route of transmission of infection is more common. This is secondary to lack of use of aseptic measures during delivery, lack of prophylactic medication in neonates, and inadequate treatment in pregnant mothers.

In one study, Singer et al. [13] reported noncontrast FLAIR sequences to be superior to post contrast T1W1. The reason for the difference in observation is most likely that the diagnosis of meningitis on FLAIR depends on the CSF protein concentration, as we have mentioned earlier, so it is possible that difference in the CSF protein concentration could have made FLAIR more sensitive. In studies which concluded that contrast-enhanced T1WI are better than FLAIR, it could have been because of less protein concentration in the CSF of their patients. Other reasons could be different imaging parameters, different MRI machines with different specifications, and different sample sizes.

A study done in 1997 by Tsuchiya and his fellows [14] compared non-contrast-enhanced FLAIR with postcontrast T1W sequence for the diagnosis of brain abscesses, meningitis, cysticercosis, and epidural empyema and concluded that noncontrast fast FLAIR images showed pathologic changes in intracranial infections, better than or as well as conventional T2- and proton density-weighted spin-echo sequences. However, postcontrast T1-weighted spin-echo sequences resulted in better visibility of abscess, meningitis, cysticercosis, and epidural empyema than did FLAIR images. However, it was noteworthy that the cisternal lesions in the patient with tuberculous meningitis were more conspicuous on FLAIR images than on T2 or proton density-weighted images, as they appeared hyperintense relative to CSF on FLAIR images.

Falzone et al. [15] published a study on contrast-enhanced fluid-attenuated inversion recovery versus contrast-enhanced spin-echo T1-weighted brain imaging. Their results show superiority of contrast-enhanced FLAIR images in comparison with contrast-enhanced spin-echo T1-weighted images in detecting enhancing brain lesions. The results are comparable to our study. However, exact comparison cannot be made as their diagnosis included enhancing brain lesions, which encompasses diseases other than meningitis.

In a research conducted by Galassia et al. [16], they showed that abnormal meningeal enhancement was positive in 35 contrast-enhanced T1-weighted MR images with Fat Saturation and in 33 contrast-enhanced FLAIR studies. They concluded that contrast-enhanced T1-weighted MR imaging with Fat Saturation is superior to contrast-enhanced FLAIR imaging in most cases for depicting intracranial meningeal diseases. The results are not comparable to our study. The reason appears to be the small sample size. The total number of patients in their study was only 24. Thirty-five examinations were done in these twenty-four patients. However, in our study, 57 patients were included in the study.

One study by Ercan N and his group concluded that postcontrast FLAIR imaging is a valuable adjunct to postcontrast T1W imaging. Precontrast and postcontrast FLAIR imaging effectively delineate parenchymal metastases, particularly leptomeningeal-cisternal and cranial-nerve metastases [17] (Table 1).

FLAIR sequences confirmed an increased CSF signal in most of the patients when obtained after I/V contrast injection. In all patients with pathologies leading to a breakdown of blood-brain barrier or neovascularization close to the SAS and ventricles, an increased signal of CSF was shown after gadolinium injection [21].

Finally, the results of our study encourage the use of postcontrast FLAIR MRI to confirm the diagnosis of meningitis, because of its high sensitivity and specificity.

## 5. Conclusion

Contrast-enhanced FLAIR sequence is more sensitive and specific than contrast-enhanced T1W sequence in the diagnosis of meningitis. Hence, contrast enhanced FLAIR sequence should be included as a routine sequence in suspected cases of meningitis for making noninvasive diagnosis.

## Figures and Tables

**Table 1:** Comparison between this study and other studies highlighting the importance of postcontrast FLAIR in the diagnosis of meningitis.

Studies	Postcontrast FLAIR	Postcontrast T1W	Noncontrast FLAIR	Postcontrast T1W FAT SAT
This study	More sensitive	Less sensitive	—	—
Allesandra et al. (2005) [11]	More sensitive	Less sensitive	—	—
Parmar et al. (2006) [7]	Similar sensitivity but higher specificity	Similar sensitivity but lower specificity	—	—
Singer et al. (1998) [13]	—	Less sensitive than noncontrast FLAIR	More sensitive than postcontrast T1W	—
Tsuchiya et al. (1997) [14]	—	Better than noncontrast FLAIR	Better than conventional T2 or proton density	—
Falzone et al. (2008) [15]	More sensitive than postcontrast T1W in enhancing parenchymal lesions	Less sensitive than postcontrast T1W in enhancing parenchymal lesions	—	—
Galassia et al. (2005) [16]	Less sensitive than postcontrast T1W FAT SAT.	—	—	More sensitive than contrast-enhanced FLAIR
Ercan et al. (2004) [17]	Postcontrast FLAIR is a valuable adjunct to postcontrast T1W	Postcontrast T1W is essential.	—	—
